# Predicting surgical outcome in patients with glioblastoma multiforme using pre-operative magnetic resonance imaging: development and preliminary validation of a grading system

**DOI:** 10.1007/s10143-017-0817-0

**Published:** 2017-02-15

**Authors:** Hani J. Marcus, Sophie Williams, Archie Hughes-Hallett, Sophie J. Camp, Dipankar Nandi, Lewis Thorne

**Affiliations:** 1 The Hamlyn Centre, Institute of Global Health Innovation, Imperial College, London, UK; 2 Department of Neurosurgery, Charing Cross Hospital, Imperial College Healthcare NHS Trust, London, UK; 3 Clinical Research Fellow and Specialty Registrar in Neurosurgery, Hamlyn Centre, Imperial College London and Imperial College Healthcare NHS Trust, Paterson Building (Level 3), Praed Street, London, W2 1NY UK; 4 Department of Medicine, Imperial College, London, UK; 5 Department of Neurosurgery, National Hospital for Neurology and Neurosurgery, UCLH Foundation Trust, London, UK

**Keywords:** Glioblastoma multiforme, Magnetic resonance imaging, Surgical outcome, Complications, Resection

## Abstract

The lack of a simple, objective and reproducible system to describe glioblastoma multiforme (GBM) represents a major limitation in comparative effectiveness research. The objectives of this study were therefore to develop such a grading system and to validate it on patients who underwent surgical resection. A systematic review of the literature was performed to identify features on pre-operative magnetic resonance imaging (MRI) that predict the surgical outcome of patients with GBM. In all, the five most important features of GBM on pre-operative MRI were as follows: periventricular or deep location, corpus callosum or bilateral location, eloquent location, size and associated oedema. These were then used to develop a grading system. To validate this grading system, a retrospective cohort study of all adult patients with supratentorial GBM who underwent surgical resection between the 1 January 2014 and the 31 June 2015 was performed. There was a substantial agreement between the two neurosurgeons grading GBM (Cohen’s *κ* was 0.625; standard error 0.066). High-complexity lesions were significantly less likely to result in complete resection of contrast-enhancing tumour than low-complexity lesions (50.0 versus 3.4%; *p* = 0.0007). The proposed grading system may allow for the standardised communication of anatomical features of GBM identified on pre-operative MRI.

## Introduction

The surgical management of patients with glioblastoma multiforme (GBM) remains contentious with a paucity of high-quality evidence to guide decision making. Nonetheless, the nihilism associated with GBM is gradually diminishing. A randomised study concluded that, even in the elderly, surgical resection was superior to biopsy alone [43]. More recently, cohort studies have found that complete resection of enhancing tumour results in prolonged survival [27, 29, 34, 39]. However, in the largest prospective study on patients with GBM, complete resection was only achieved in approximately a third of patients, suggesting difficulty in defining marginal, enhancing tumour intra-operatively using conventional microsurgical techniques [38].

A multitude of surgical innovations have been introduced to maximise the resection of GBM including fluorescence-guided surgery and various other intraoperative imaging techniques [3, 20, 26]. Over the next decade, emerging technologies such as confocal laser endomicroscopy, rapid evaporative ionisation mass spectrometry and Raman spectroscopy are expected to further expand the surgical armamentarium [2, 19, 24].

Selecting from the aforementioned array of surgical innovations is difficult. The operative techniques used for the resection of GBM have historically been based on a surgeon’s training and experience and the local availability of resources. The Balliol Collaboration has proposed the Idea, Development, Exploration, Assessment and Long-term follow up (IDEAL) model for surgical innovation; the central tenet being that innovation and evaluation should proceed together [13, 17, 31]. Evaluation of surgical innovations that maximise the resection of GBM is especially challenging because of the heterogeneity in tumour anatomy and the complexity of surgical resection.

In other cerebral pathologies such as arteriovenous malformation (AVM), several anatomical features on pre-operative imaging have been found to predict surgical outcome including size, relationship with eloquent structures and pattern of venous drainage [37]. The lack of a similarly simple, objective and reproducible system to describe GBM represents a major limitation in comparative effectiveness research. The aims of this study were therefore to (1) identify features on standard pre-operative magnetic resonance imaging (MRI) that predict the surgical outcome of patients with GBM and use these to develop a grading system and (2) validate this grading system on patients who have underwent surgical resection.

## Materials and methods

### Development

A systematic review of the literature was performed to identify features on pre-operative MRI that predict the surgical outcome of patients with GBM. The Preferred Reporting Items for Systematic Reviews and Meta-Analyses (PRISMA) statement was used in the preparation of this section of the manuscript [32].

#### Inclusion and exclusion criteria

Articles that (1) featured adult patients (greater than 18 years of age) with supratentorial GBM undergoing surgical resection and (2) reported on the use of pre-operative MRI to predict residual disease or survival were included. Articles that used advanced imaging techniques such as segmentation and volumetric analysis, which are not widely available, were excluded.

#### Information sources, search strategy and study selection

The PubMed database was searched between 1 January 1995 and 31 June 2015 using the search terms (glioblastoma OR “malignant glioma” OR “high grade glioma” OR “high-grade glioma”) AND (pre-operative OR preoperative OR preop OR pre-op) AND (prediction OR predictive OR scoring OR score) AND (outcome OR resection OR resectability OR “progression free survival” OR PFS OR “overall survival” OR OS).

Titles and abstracts were screened to identify articles that met appeared to meet the inclusion criteria (HJM and AHH; both clinical research fellows). Full papers were then retrieved for detailed review. Discrepancies were resolved by consensus and discussion with the senior author.

#### Data collection process and data items

The following data were extracted from selected articles (HJM and AHH): (1) study design, (2) patient characteristics, (3) pre-operative MRI features, (4) surgical outcomes and (5) findings. When both univariate and multivariate analyses were presented, we preferentially included the more significant findings. Corresponding authors were contacted to provide supplemental data when required.

#### Development of grading system

The pre-operative MRI features most frequently used to predict surgical outcomes were used to develop a grading system. Features were selected such that the proposed grading system made clinical sense and could be easily incorporated into practice using a standard contrast-enhanced T1-weighted MRI.

### Validation

A retrospective cohort study was performed to validate the grading system. The Strengthening the Reporting of Observational Studies in Epidemiology (STROBE) statement was used in the preparation of this section of the manuscript [42].

#### Setting and participants

The study was conducted at Charing Cross Hospital, which acts as regional referral centre for brain tumours in North West London. The centre comprises three specialist neurosurgeons, who spend at least half of their clinical programmed activity in neurooncological surgery.

All referrals were recorded on a prospectively maintained database. The database was searched between the 1 January 2014 and the 31 June 2015 to identify all adult patients with supratentorial GBM who underwent craniotomy and resection. Patients who underwent a burr hole and stereotactic biopsy only were excluded.

#### Variables and data sources

All patients with GBM received therapy according to the National Institute of Clinical Excellence (NICE) guidance, including the following: high-dose dexamethasone, discussion of their case in a dedicated neurooncology multidisciplinary meeting, pre-operative MRI with contrast, image-guided craniotomy and microsurgical resection and post-operative MRI with contrast within 72 h of surgery. Intraoperative ultrasound was available but was used according to surgeon preference.

The pre-operative contrast-enhanced T1-weighted MRI scans were graded by two neurosurgeons blinded to the outcome. The post-operative contrast-enhanced T1-weighted MRI scan was evaluated by a consultant neuroradiologist blinded to the grade to determine the extent of resection (complete resection of all contrast-enhancing tumours or not). All images were reviewed on standard display monitors.

A retrospective case note review was also performed to identify any immediate surgical complications, which were recorded according the Clavian-Dindo classification [12, 15]. The strength of this classification system is that it largely relies on the therapy used to treat the complication, which are easily identified in retrospective analyses.

#### Study size and statistical methods

It was estimated using pilot data that the previous criteria would identify approximately 100 patients. Based on similar studies validating grading systems in other cerebral pathologies, we considered 100 patients sufficient for meaningful analysis [37].

Data were analysed using with SPSS v 20.0 (IBM, Illinois, USA). The mean and standard deviation were calculated for parametric variables, and the median and interquartile ranges were calculated for non-parametric variables. Cohen’s *κ* was calculated to determine the agreement between the two neurosurgeons grades. The chi-square test was then used to (1) compare the grade against extent of resection and (2) compare the grade against the presence of major complications (Clavian-Dindo greater than 3a). A value of *P* < 0.05 was considered statistically significant.

## Results

### Development

#### Study selection

**Fig. 1:**
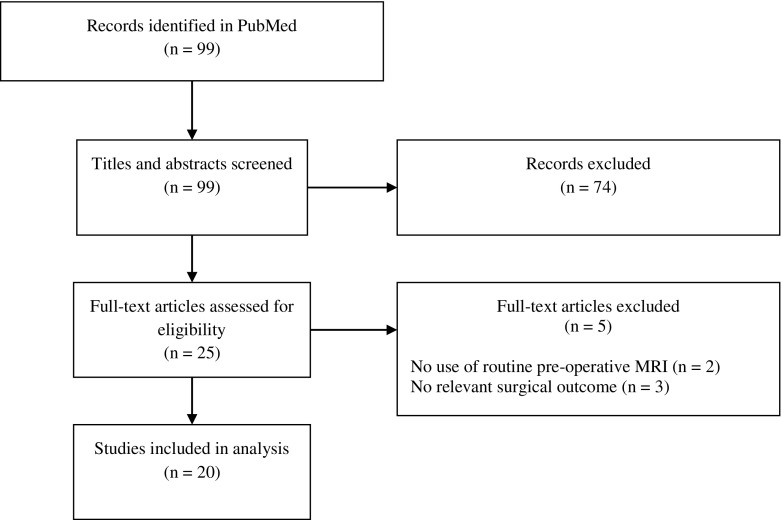
Selection of articles that identify features on pre-operative MRI that predict the surgical outcome of patients with GBM

In all, 99 article titles and abstracts were screened, 25 full articles were considered relevant and further assessed for their eligibility, and 20 articles were ultimately included (Fig. 1).

#### Study characteristics

The included studies comprised one prospective cohort study, 18 retrospective cohort studies and one case-control study. The patient characteristics, pre-operative MRI features, surgical outcomes and findings are summarised in Table 1.

**Table 1:** Summary of study characteristics

First author (year)	Study design	Patients characteristics	Pre-operative MRI features	Surgical outcomes	Findings
[1]	Case-control study	*n* = 100 with GBM; 50 pts with long OS, 50 pts with short OS.	Multifocal disease Laterality Periventricular (subventricular zone) location	OS	OS reduced if both hemispheres involved (*p* = 0.074, univariate) OS reduced with periventricular location (*p* = 0.038, univariate)
[4]	Retrospective cohort study	*n* = 54 with GBM	Volume Periventricular location	EOR	EOR reduced with increased volume (*p* < 0.05, univariate) EOR reduced with periventricular location (*p* < 0.05, univariate)
[6]	Retrospective cohort study	*n* = 52 with GBM; 26 pts with periventricular location, 26 pts with other location.	Diameter Periventricular location	OS	OS reduced with periventricular location (*p* = 0.02, univariate)
[7]	Retrospective cohort study to construct and validate scoring system	*n* = 393	Brain lobe Diameter Periventricular location Eloquent location	OS	OS reduced with periventricular location (*p* = 0.04, multivariate)
[8]	Retrospective cohort study	*n* = 129 with GBM; aged >65 years.	Brain lobe Diameter Eloquent location	OS	OS reduced with increased diameter (*p* = 0.002, multivariate)
[9]	Multicentre retrospective cohort study to validate scoring system	*n* = 334 with GBM	Periventricular location	OS	OS reduced with periventricular location (*p* = 0.04, multivariate)
[10]	Retrospective cohort study	*n* = 100 with GBM; KPS <60.	Diameter Brain lobe Periventricular location	OS	OS increased with increased diameter > 2 cm (*p* = 0.01, multivariate)
[11]	Retrospective cohort study	*n* = 233 with glioma; 121 with GBM.	Corpus callosum involvement	EOR PFS OS	EOR, PFS and OS reduced with corpus callosum involvement (*p* < 0.001, *p* = 0.006 and *p* = 0.011 respectively; multivariate)
[16]	Retrospective cohort study	*n* = 46 with GBM	Brain lobe Diameter	OS	OS reduced with temporal location (*p* = 0.0130, multivariate) OS reduced with increased diameter (*p* = 0.0395, multivariate)
[21]	Retrospective cohort study	*n* = 48 with GBM	Volume Necrosis Contrast enhancement Associated oedema	OS	OS reduced with necrosis (*p* < 0.001, multivariate) OS reduced with enhancement (*p* = 0.003, multivariate) OS reduced with oedema (*p* < 0.004, multivariate)
[22]	Retrospective series	*n* = 516 with GBM	Laterality	OS	OS reduced if both hemispheres involved (*p* < 0.01, multivariate)
[23]	Retrospective cohort study	*n* = 45 with GBM	NER:CER Area of NER Definition of margins Laterality Deep white matter involvement Associated oedema	OS PFS	NER crossing the midline (OS *p* = 0.0125, PFS *p* = 0.0661, multivariate).
[25]	Prospective cohort study	*n* = 80 with HGG; aged 60–83 years.	Volume Eloquent location	OS	No significant associations found.
[27]	Retrospective cohort study	*n* = 420 with GBM	Necrosis Contrast enhancement Deep location Eloquent location Associated oedema Midline shift	OS	OS reduced with necrosis (*p* = 0.01, multivariate) OS reduced with contrast enhancement (*p* = 0.02, multivariate) OS reduced with eloquent location (*p* = 0.02, univariate) OS reduced with oedema (*p* = 0.04, univariate)
[28]	Retrospective cohort study	*n* = 116 with GBM	Laterality Brain lobe Diameter Cysts Contrast enhancement Deep or eloquent location Associated oedema Midline shift	PFS OS	PFS was dependent upon brain lobe (*p* = 0.001, multivariate) OS reduced with deep or eloquent location (*p* = 0.009, multivariate)
[30]	Retrospective cohort study	*n* = 205 with GBM	Deep or eloquent location	OS	OS reduced with deep or eloquent location (*p* < 0.0001, multivariate)
[33]	Multicentre retrospective cohort study to construct and validate scoring system	*n* = 143 with recurrent GBM; 34 pts to devise scoring system and 109 pts to validate.	Brain lobe Laterality Volume Eloquent location	OS	OS reduced with greater volume (*p* < 0.001, univariate) OS reduced with eloquent location (*p* < 0.001, univariate)
[35]	Multicentre retrospective cohort study	*n* = 110 with GBM	Diameter Associated oedema	OS	OS reduced with associated oedema (*p* = 0.006, multivariate)
[39]	Retrospective cohort study (using prospectively collected trial data)	*n* = 243 with GBM; 121 pts received 5-ALA, 122 pts did not.	Volume Hemisphere Periventricular location Eloquent location Associated oedema Midline shift	EOR OS	EOR reduced with eloquent location (*p* = 0.0231, univariate)
[41]	Retrospective cohort study	*n* = 65 with GBM; received 5-ALA.	Brain lobe Volume Periventricular location	OS	OS reduced with periventricular location (*p* = 0.008, multivariate)

*pts* patients, *GBM* glioblastoma multiforme, *HGG* high-grade glioma, *EOR* extent of resection, *PFS* progression-free survival, *OS* overall survival, *KPS* Karnofsky Performance Status score, *CER* contrast-enhancing region, *NER* non-enhancing region

#### Grading system

Five features on pre-operative MRI were identified that were found to be predictive of surgical outcome in at least two studies (Table 2): periventricular or deep location, corpus callosum or bilateral location, eloquent location, size and associated oedema. These features were used to develop a grading system for adult patients with supratentorial GBM (Table 3).

**Table 2:** Summary of pre-operative features that predict surgical outcome

Pre-operative MRI feature	Number of studies that analysed	Number of studies that found feature predictive of outcome
Periventricular or deep location^a^	12	8
Eloquent location	8	5
Size^b^	13	5
Associated oedema	6	3
Corpus callosum involvement or bilateral location^c^	6	2

Only features with >2 studies supporting their use were included

^a^Periventricular and deep location were combined

^b^Diameter and volume were combined

^c^Corpus callosum and bilateral location were combined

**Table 3:** The proposed grading system for adults with supratentorial GBM

Pre-operative MRI feature	Score
Periventricular or deep location	
≥10 mm from ventricle	0
<10 mm from ventricle	1
Corpus callosum or bilateral location	
No corpus callosum involvement	0
Corpus callosum involvement or bilateral location	1
Eloquent location	
Not eloquent location	0
Eloquent location (motor or sensory cortex, language cortex, insula or basal ganglia)	1
Largest diameter of tumour (mm)	
<40	0
≥40	1
Associated oedema	
<10 mm from contrast-enhancing tumour	0
≥10 mm from contrast-enhancing tumour	1
Total	0–5 0–1 Low complexity 2–3 Moderate complexity 4–5 High complexity

All features are assessed using the pre-operative contrast-enhanced T1-weighted MRI

**Fig. 2:**
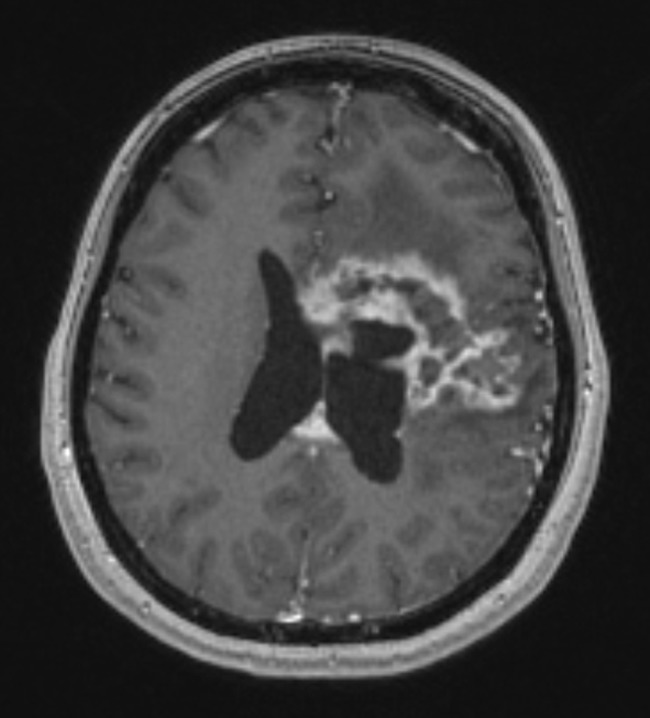
Example of a high-complexity lesion (5 points) on pre-operative contrast-enhanced T1-weighted MRI

The grading system was designed to be simple, objective and reproducible. Each feature is measured on a standard pre-operative contrast-enhanced T1-weighted MRI: periventricular or deep location if the contrast-enhancing tumour is located within 10 mm of the ventricles; corpus callosum or bilateral location if contrast-enhancing tumour extends into these regions; eloquent if contrast-enhancing tumour extends into motor or sensory cortex, language cortex, insula or basal ganglia; large if the diameter of the contrast-enhancing tumour exceeds 40 mm; and associated oedema if hypo-intensity extends more than 10 mm from contrast-enhancing tumour. All features are weighted equally, with one point assigned if a feature was present and no points if absent. The sum of these features is used to describe lesions as low (0–1 points), moderate (2–3 points) and high complexity (4–5 points). An example of a high-complexity tumour is illustrated in Fig. 2.

The grading system uses a standard pre-operative contrast-enhanced T1-weighted MRI as such imaging is readily available, allowing the grading system to be used in resource-limited settings and in retrospective studies. The radiological definitions of periventricular or deep location, large diameter and associated oedema were drawn from the literature and, where there was discrepancy, using the expert opinion of the senior authors (DN and LT). All features were weighted equally, and the sum of these features is used to describe lesions as low, moderate and high complexities, to ensure that the grading system is as simple as possible and to increase the statistical power for series comparisons.

### Validation

#### Participants and descriptive data

In all, 106 patients were identified with supratentorial GBM who underwent craniotomy and resection. Of these, 18 patients were excluded because their imaging (*n* = 9) or clinical notes (*n* = 9) could not be found, and 88 patients were included. The patient demographics are summarised in Table 4.

**Table 4:** Patient demographics

Sex	
- male/female	1.9:1
Age median	
- (interquartile range)	59 years (46–70)
GBM	
- primary: recurrent	7:1

#### Outcome data and main results

Pre-operative MRI features are summarised in Table 5. The majority of tumours had a periventricular location (77.3%; 68/88), were greater than 40 mm in diameter (70.5%; 62/88) and had significant associated oedema (69.3%; 61/88). Corpus callosum involvement, or bilateral location, was seen in 33.0% of tumours (29/88), while eloquent location involvement was seen in 43.2% (38/88) of tumours.

**Table 5:** Features as assessed using the pre-operative contrast-enhanced T1-weighted MRI

Pre-operative MRI feature	Number
Periventricular or deep location	
≥10 mm from ventricle	20 (22.7%)
<10 mm from ventricle	68 (77.3%)
Corpus callosum or bilateral location	
No corpus callosum involvement	59 (67.0%)
Corpus callosum involvement or bilateral location	29 (33.0%)
Eloquent location	
Not eloquent location	50 (56.8%)
Eloquent location (motor or sensory cortex, language cortex, insula or basal ganglia)	38 (43.2%)
Largest diameter of tumour (mm)	
<40	26 (29.5%)
≥40	62 (70.5%)
Associated oedema	
<10 mm from contrast-enhancing tumour	27 (30.7%)
≥10 mm from contrast-enhancing tumour	61 (69.3%)
Total	88

Post-operative MRI demonstrated complete resection of contrast-enhancing tumour in 15 patients (17.0%). Three patients (3.4%) had major complications: two had intracerebral haemorrhage, and one had massive pulmonary embolism.

**Fig. 3:**
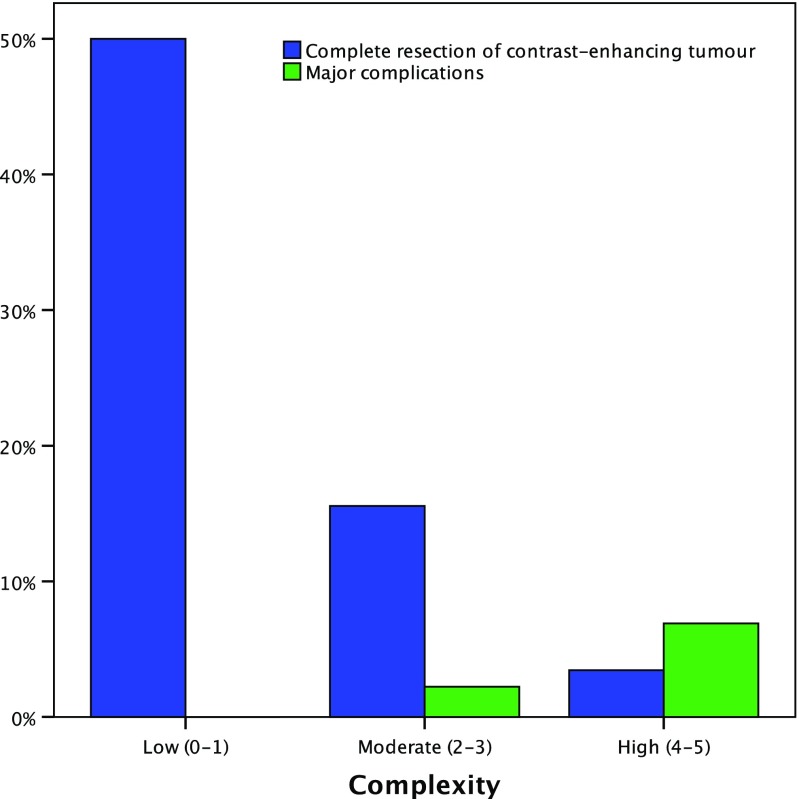
Grade and surgical outcome

There was a substantial agreement between the two neurosurgeons grading GBM (Cohen’s *κ* was 0.625; standard error 0.066). The grades and surgical outcomes are summarised in Table 6 and Fig. 3. There was a significant association between grades and extent of resection (*p* = 0.0007) but not complications (*p* = 0.4148).

**Table 6:** Grade and surgical outcome

Grade	Number	Complete resection of contrast-enhancing tumour	Major complications
Low complexity(0–1 points)	14	7 (50.0%)	0 (0%)
Moderate complexity(2–3 points)	45	7 (15.6%)	1 (2.2%)
High complexity(4–5 points)	29	1 (3.4%)	2 (6.9%)
Total	88	15 (17.0%)	3 (3.4%)

## Discussion

### Principal findings

Currently, the literature on GBM management contains limited data on relevant surgical anatomy. When such data are available, it is often described in a variable manner, making comparative effectiveness research difficult. This study reports the development and validation of a simple, objective and reproducible grading system for GBM. The proposed grading system allows for the standardised reporting of the five most important features of GBM on pre-operative MRI: periventricular or deep location, corpus callosum or bilateral location, eloquent location, size and associated oedema. Moreover, this grading system was found to be predictive of surgical outcome. High-complexity lesions were significantly less likely to result in complete resection of contrast-enhancing tumour than low-complexity lesions (50.0 versus 3.4%; *p* = 0.0007). High-complexity lesions were also more likely to result in major complications, though this did not achieve statistical significance (6.9 versus 0%; *p* = 0.4148).

### Comparison with other studies

Each element of the proposed grading system has been previously identified in the literature as affecting surgical outcome. The most consistently reported of these elements is GBM near the ventricles, or deep location, which eight articles found to be associated with reduced extent of resection and overall survival. The resection of deep-seated GBM necessitates pial and subpial transection. Multiple studies have demonstrated that lesions of the deep white matter tracts elicit more severe and permanent neurological deficits than cortical injuries of comparable volume, making the complete resection of contrast-enhancing tumour challenging [18]. Benveniste et al. found that, for tumours under 30 ml in volume, only 77% of those lying adjacent to the ventricles were completely resected, compared to 91% of those lying elsewhere. Emerging technologies such as high-definition fibre tractography and endoscopic port surgery may improve the visualisation and resection of tumours lying near the ventricles [5, 18, 40].

The presence of cerebral oedema associated with GBM is a common feature on pre-operative MRI and is known to affect overall survival. All the patients in our cohort were given high-dose dexamethasone pre-operatively, and in more than two thirds, major oedema persisted on imaging. Major oedema can result in considerable difficulty during surgical resection, obscuring identification of anatomical landmarks and making brain tissue less amenable to manipulation. To this end, evidence suggests that minimal oedema is associated with increased overall survival (albeit only with univariate analysis), while major oedema, extending more than 10 mm from the tumour margin, is associated with reduced survival [21, 27, 35].

It has long been recognised that individual tumour cells frequently spread into the contralateral hemisphere in patients with GBM [14]. However, only a third of patients in our cohort were found to have contrast-enhancing tumour infiltrate the corpus callosum on pre-operative MRI. The surgical resection of lesions within the corpus callosum, particularly the posterior region, can result in major complications such as disconnection syndrome. Infiltration of the corpus callosum is therefore associated with significantly reduced extent of resection, reduced progression-free survival and reduced overall survival [11]. Similarly, tumours extending into both hemispheres are associated with worse overall survival [1, 22].

Larger GBM, assessed by both linear and volumetric parameters, is associated with reduced extent of resection and worse overall survival [4, 8, 10, 16, 33]. A number of metrics have been used to dichotomise tumours according to size, including a tumour volume greater than 30 ml or a maximal diameter greater than 2 or 40 mm [4, 7, 10]. As volumetric and linear metrics have been shown to be comparable and linear metrics are more straightforward to measure on standard MRI, we used a maximal diameter greater than 40 mm in the proposed grading system.

Several studies have identified GBM located within eloquent regions as a negative predictor of extent of resection and overall survival [16, 27, 28, 30, 33, 39]. The regions considered eloquent vary in the literature, but those most commonly included were used in the proposed grading system (motor or sensory cortex, language cortex, insula or basal ganglia). Historically, complete resection of lesions residing within these regions was believed to invariably result in permanent neurological deficits. Recent evidence, however, points to considerable plasticity within these regions, and techniques such as awake craniotomy may yet allow for complete resection of contrast-enhancing tumours in selected cases [40].

Taken together, the five aforementioned pre-operative MRI features have a major impact on surgical outcome. Studies comparing different operative techniques must therefore carefully account for these confounders. Although the overall rate of complete resection in our preliminary validation study was modest compared to the literature (17.0%; 15/88), the rate of complete resection of contrast-enhancing tumour varied widely from 3.4% in high-complexity lesions (4–5 features) to 50.0% in low-complexity lesions (0–1 features). In the largest prospective study on patients with GBM, only 36% of patients were found to have their complete resection of contrast-enhancing tumour using conventional microsurgical techniques [38]. Other studies, which report a rate of complete resection of contrast-enhancing tumour in up to 90% of cases, are usually retrospective, often exclude patients in whom complete resection is deemed impossible such as those involving the corpus callosum or eloquent brain, and may combine near total and gross total resection in their analyses [36].

To the best of our knowledge, the proposed grading system is the first to use features on pre-operative MRI to predict the surgical outcome of adult patients undergoing craniotomy for GBM. However, several other systems have been described that use a combination of demographic, clinical and radiological features to predict overall survival. Chaichana et al. developed a system that included the patient’s Karnofsky Performance Status, age, the presence of a motor deficit, the presence of a language deficit and periventricular tumour location. This system was initially validated on 393 prospective cases at one centre and then later on 334 patients across three centres [10]. Park et al. developed a similar system using the Karnofsky Performance Status, tumour volume and tumour location within eloquent or critical regions, which was validated on a retrospective cohort of 34 consecutive patients at one centre [33]. Although these systems are useful, the proposed grading system differs in focusing on anatomical features of GBM that influence the operative complexity and therefore the extent of resection and likelihood of major complications.

### Limitations

There were several limitations to the validation of the proposed grading system. First, the retrospective nature of the study introduces the possibility of, for example, selection bias. The accurate and precise recording of patient complications, in particular, is difficult in such study designs. Second, the single-centre design makes it difficult to generalise the findings to different centres with different patient populations, case selection and operative techniques. Third, the study was powered to validate the grading system with respect to extent of resection, but not major complications. Finally, the study did not measure important other surgical outcomes such as progression-free survival and overall survival. These limitations might be addressed through prospective, multicentre and larger studies.

## Conclusions

The proposed grading system may allow for the standardised communication of anatomical features of GBM identified on pre-operative MRI. While the grading system may not capture all the elements that contribute to the complexity of individual cases, we believe that it captures the most relevant characteristics in a reproducible manner. We hope that use of this grading system in clinical practice and in the literature will enable more standardisation of clinical care and more meaningful comparisons of clinical studies.

### Author contributions

HJM and AHH were involved in the study conception, acquisition of data, analysis of data and drafting of the manuscript. SW and SJC were involved in the acquisition of data, analysis of data and drafting of the manuscript. DN and LT were involved in the study conception and critical revision of the manuscript.
